# What’s Wrong in a Jump? Prediction and Validation of Splice Site Variants

**DOI:** 10.3390/mps4030062

**Published:** 2021-09-05

**Authors:** Giulia Riolo, Silvia Cantara, Claudia Ricci

**Affiliations:** Department of Medical, Surgical and Neurological Sciences, University of Siena, 53100 Siena, Italy; giulia.riolo@gmail.com (G.R.); cantara@unisi.it (S.C.)

**Keywords:** alternative splicing, splicing sites, splice variant, prediction tools, machine learning, experimental validation, variant classification

## Abstract

Alternative splicing (AS) is a crucial process to enhance gene expression driving organism development. Interestingly, more than 95% of human genes undergo AS, producing multiple protein isoforms from the same transcript. Any alteration (e.g., nucleotide substitutions, insertions, and deletions) involving consensus splicing regulatory sequences in a specific gene may result in the production of aberrant and not properly working proteins. In this review, we introduce the key steps of splicing mechanism and describe all different types of genomic variants affecting this process (splicing variants in acceptor/donor sites or branch point or polypyrimidine tract, exonic, and deep intronic changes). Then, we provide an updated approach to improve splice variants detection. First, we review the main computational tools, including the recent Machine Learning-based algorithms, for the prediction of splice site variants, in order to characterize how a genomic variant interferes with splicing process. Next, we report the experimental methods to validate the predictive analyses are defined, distinguishing between methods testing RNA (transcriptomics analysis) or proteins (proteomics experiments). For both prediction and validation steps, benefits and weaknesses of each tool/procedure are accurately reported, as well as suggestions on which approaches are more suitable in diagnostic rather than in clinical research.

## 1. Introduction

How many protein coding genes have been described in humans? The answer is approximately 25,000–30,000. This exorbitant number is nothing when compared with the almost 90,000 different proteins that form human proteome. This phenomenon can be possible thanks to mechanisms of alternative splicing (AS), a process that was first proposed by Gilbert in 1978 [1]. AS is crucial to enhance gene expression, to drive cellular differentiation and organism development. More than 95% of human genes have been found to undergo alternative splicing in a developmental, tissue-specific or signal transduction-dependent way [2]. During AS, exons, or portions of exons or noncoding regions within a pre-messenger RNA (pre-mRNA) transcript, are differentially fixed or skipped, resulting in multiple protein isoforms [3]. Regulation of alternative splicing is complex with several elements interacting in a coordinated manner including cis-acting and trans-acting factors, spliceosome components as well as chromatin or RNA structure together with the presence of alternative transcription initiation (ATI) or alternative transcription termination (ATT) sites [3].

In addition, the presence of genomic variants, involving consensus splicing regulatory sequences in different parts of a gene, may modify the splicing process, alter the mRNA and eventually affect the corresponding protein-coding sequence [4].

The estimate of variant impact on RNA processing is not always simple, and can lead to improper variant classification. The aim of this review is to provide an updated approach to this challenge. In the first part, we describe the key element involved in pre-mRNA maturation and the potential consequences of genomic variant on the splicing process. Then, we review the main computational tools that allow identifying and characterizing genomic variants that may alter the splicing process. Particular attention is paid to the Machine Learning (ML) approach. We discuss the main strengths and weaknesses of the different approaches, to enable the researchers to estimate and choose the right tool/s for their purposes. In the second part, the experimental methods to validate the in silico predicted splicing variants are described, suggesting which approaches are more suitable in diagnostic rather than in clinical research.

## 2. Constitutive Splicing vs. Alternative Splicing

**Figure 1:**
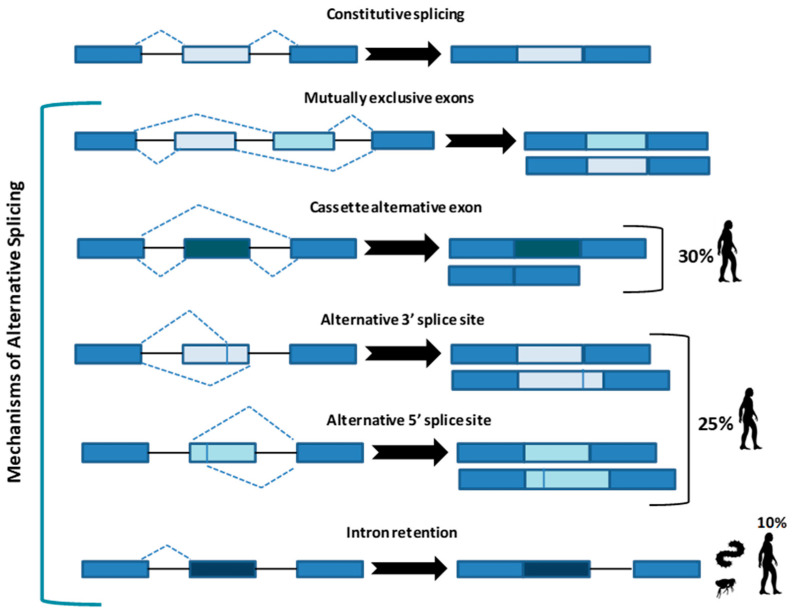
Constitutive splicing and the five main types of alternative splicing. Cassette alternative exon and the alternative 3’ or 5’ splice site are the most common in humans (30% and 25%, respectively), while intron retention is typical of metazoans and less present in humans (10%). Arrows indicate the resulted sequence after intron/exon removal.

Whatever the mechanism, the final goal of splicing is to remove introns from a protein-coding RNA to generate a mature mRNA to produce a functional protein. Constitutive splicing follows the order in which exons are in the gene, whereas AS represents a variation from this preferred sequence where some exons are skipped, producing a variety of mature mRNA and thus different proteins. At least five strategies (Figure 1) of AS have been described.

**Figure 2:**
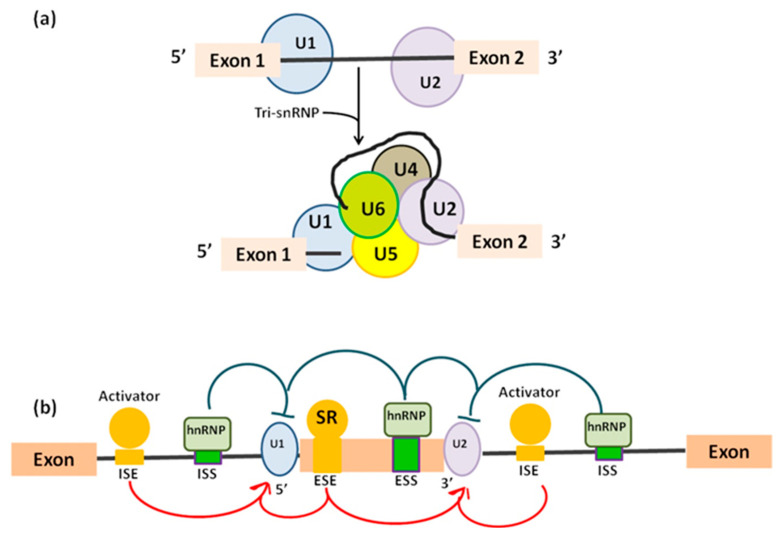
(**a**) U1 binds to exon 1 and U2 binds to exon 2 in order to define 5’ ends of the intron before removal. Addition of tri-snRNP U4/U6.U5 determines the full spliceosome assembly in humans. (**b**) Role of cis- and trans-regulatory sequences during alternative splicing. Cis-regulatory elements are located in the alternatively spliced exon or in its flanking introns. Cis-factors positively modulate intronic/exonic splicing enhancers (ISE/ESE) and negatively regulate intronic/exonic splice silencer (ISS/ESS). Cis-sequences are bound by trans-factors such as serine/arginine (SR) proteins or the heterogeneous nuclear ribonucleoprotein (hnRNP).

In the “mutually exclusive exons”, one out of two exons (or one group out of two exon groups) is maintained, while the other one is spliced out [5]. In the “cassette alternative exon”, which represents the most common mechanism in vertebrates (30% in humans) and invertebrates, an exon may be spliced out of the primary transcript or retained [3]. The “alternative 3’ or 5’ splice site” (25% of AS in humans) can produce two splice transcripts: one contains the extension and the other excludes it. These transcripts can be formed in different ratios, one can be more abundant compared with the other. If an alternative 3’ splice acceptor site is used, we observed a change of the 5’ boundary of the downstream exon. When an alternative 5’ splice site is used, the 3’ boundary of the upstream exon is changed [6]. Finally, in “intron retention”, which is the preferred mechanism by lower metazoans and represents 10% of AS in humans [7], an intron sequence may be spliced out or retained. The retained sequence is not flanked by introns. In humans, all these steps of intron excision and exons ligation, are carried out by the spliceosome complex, a large ribonucleoprotein machinery in which more than 300 proteins assemble in sequence with the uridine-rich small nuclear RNA molecules (U snRNAs) to form individual small nuclear ribonucleoprotein complexes (snRNPs). In human nuclei, the majority of splicing reactions are carried out by U1, U2, U3 snRNPs, and U4/U6.U5 tris-snRNP [8] (Figure 2a).

Pre-mRNA is recognized by splicing machinery at conserved RNA elements: the 5’ splice site at the exon-intron border (donor site), the 3’ splice site at the intron–exon border (acceptor site), and the branch point, which is followed by a polypyrimidine track [9,10] and placed approximately 18–40 nucleotides upstream of the acceptor site [11] (Table 1). In order, donor site is recognized by U1 snRNP [12], then the U2 auxiliary factor binds to the polypyrimidine and the acceptor site [10] generating a complex called “E complex”. Next, U2 snRNP binds the branchpoint, resulting in the A complex [13]. Binding of the U4/U6.U5 tri-snRNP leads to the B complex [14], which is first activated [15].

What influences the final decision of which exons will end up in the mature mRNA? Usually shorter exon length, weaker splicing signals at different splice site or higher sequence conservation adjacent orthologues alternative exons are the main factors participating in the choice [16]. Additionally, a pivotal role in deciding exons in final mRNA is played by cis-acting elements and trans-acting factors. Cis-acting elements are short nucleotide motifs and include exonic or intronic splicing enhancer and associate with the trans-acting factor serine/arginine-rich (SR) proteins. Enhancer elements play a leading role in constitutive splicing. Similarly, also belonging to the cis-acting proteins are exonic or intronic splicing silencer which are bound by heterogeneous nuclear ribonucleoproteins (hnRNPs) negative trans-acting factors and mainly participate to alternative splicing [3] (Figure 2b). In addition to cis-regulatory sequences and their cognate trans-acting factors, alternative splicing is controlled by its coupling to RNA polymerase II (RNAPII) transcription [17]. This coupling requires the C-terminal domain (CTD) of the RNAPII largest subunit. CTD phosphorylation affects the transcriptional properties of RNAPII and the outcome of co-transcriptional AS by mediating the consequences of splicing factors and by modulating transcription elongation rates [17]. CTD takes part in gene expression-related functions from 5’ capping, splicing, polyadenylation, and chromatin remodelling, becoming a key factor in governing the interactions between transcription and splicing. To complicate the picture even more, is the existence of alternative transcription initiation (ATI) and alternative transcription termination sites (ATT) present in the 5’ UTRs and 3’ UTRs, respectively [18], which contribute to generate transcriptome diversity. It is evident that incidence and functional implication of different types of alternative events varies between functional domains of transcripts. As a result, AS is common in the 5’ UTRs and coding sequences but is rare in the 3’ UTRs given the modest intron density in this region [18]. Finally, the presence of a premature termination codon (PTC) can cause changes in the splicing pattern of a pre-mRNA. Exon skipping is common under the selective pressure of a PTC, when normally introduction of a PTC into the open reading frame of a protein-coding gene will represent a protective mechanism, leading to nonsense-mediated mRNA decay able to avoid the translation of functionally defective proteins [19].

In addition, both alternative and constitutive splicing are affected by chromatin structure, which works either by modulating the RNAPII elongation rate or by promoting the recruitment of splicing factors [20]. The resultant mature mRNA is, thus, a reflection of DNA modifications such as DNA methylation or histone modifications.

## 3. Genomic Variants Affecting Splicing Process

Considering the complexity of splicing and its role in the correct protein synthesis, any alteration of this process may cause modifications of specific mRNAs and proteins, and thus lead to aberrant cellular functions [21]. The presence of genomic variants, e.g., nucleotide substitutions, insertions and deletions, involving consensus splicing regulatory sequences in a specific gene, may modify the splicing process, cause partial or complete intron gain or exon loss from the mature mRNA and ultimately alter mRNA and corresponding protein-coding sequence [4].

Even though splicing variants may disrupt cis-acting splicing elements or involve trans-acting factor, usually the term “splicing variant” is used to refer to a mutation in the cis consensus sequences. These variants may be present in both exons and introns and lead to disruption of existing splice sites, creation of new ones, or activation of cryptic sites. They can also affect splicing enhancers and silencers or modify the mRNA secondary structure, impairing the binding of the spliceosome elements.

The typical consequence of these variants is exon or exon fragment skipping during the splicing process. When the result is an in-frame deletion, a shortened protein will be produced. Though the deletion causes the shift of the open reading frame, a premature stop codon may be created and a shorter protein may be synthesized. On the other hand, the presence of the PTC in the transcript may also result in a faster mRNA degradation. The degradation of the defective messenger RNA, which occurs through a protective process called nonsense mediated decay (NMD), prevents aberrant protein synthesis and results in the same effect as gene deletion or nonsense mutation [22].

### 3.1. DNA Variants in Canonical Splicing Sites

The “classical” definition of splicing variant refers to DNA variants affecting canonical splicing sites: splicing acceptor and donor sites, branch point adenosine, and polypyrimidine tract. Variants involving any of those sequences may alter pre-mRNA splicing, leading to exon skipping/shortening, or partial/full intron retention in the mRNA [23,24,25].

#### 3.1.1. Variants in Splicing Acceptor and Donor Sites

Variants in splicing acceptor and donor sites involve highly conserved sequences defining exon-intron boundaries and therefore may modify the interaction between pre-mRNA and splicesome complex. The most classical variants involve the +1 and +2 residues at the 5’ donor splice site and −1 and −2 residues at the 3’ acceptor splice site. These variants may cause a single exon skipping (the most frequent consequence), or lead to the occurrence of an alternative splicing site, when the presence of the variants exposes a cryptic splice site in a neighboring exon or intron. As a consequence, an intron fragment can be included or an exon fragment can be removed, depending on the position of the cryptic splice site in intron or exon, respectively [26,27].

When searching for canonical splice variants for diagnostic or research purposes, exon DNA and short neighboring intron sequences are commonly the templates for Sanger sequencing or next-generation sequencing (NGS), thus these variants are easily identified [28].

#### 3.1.2. Variants Affecting Branch Point and Polypyrimidine Tract

The branch point motif is located between −9 and −400 bp downstream from the acceptor site and in humans is characterized by the consensus sequence YUNAY. Since the sequences of the branch point are highly degenerated, their exact localization may be hard to identify; however, these sequences are crucial for the spliceosome complex formation. Variants in the branch point motif might cause an exon skipping, as a consequence of improper binding of snRNP splicing proteins and disruption of the acceptor splicing site, or lead to intron partial/total intron retention, if they create a new 3’ splice site [29].

The polypyrimidine tract is localized until 40 bp from the acceptor splice site, upstream of the branch point motif. This sequence is recognized by polypyrimidine tract-binding proteins belonging to spliceosome complex, which are involved in alternative splicing regulation. Variants in this sequence probably result in splicing alterations, even though only few of these variants have been identified so far [30].

In general, variants at the branch point and polypyrimidine tract are very rare. A possible explanation is that they are difficult to identify, since their consensus sequences are degenerated and their exact localization is hard to predict. In addition, they are not usually considered when the genomic DNA is analyzed for diagnostic purposes, and the interest is mainly focused on coding sequences.

### 3.2. Exonic Variants Affecting Splicing

In addition to canonical splice variants, also mutations in the exonic sequences may strongly affect splicing process. These exonic variants may exert a dual effect. Indeed, they can lead to modifications of pre-mRNA processing and the loss of an exon fragment, introducing a new 5’ or 3’ splice site or activating a cryptic site, which could be stronger than the original one. On the other hand, the exonic variant may disrupt an exonic splicing enhancer (ESE) causing the whole exon skipping [31].

As a result of the habit to evaluate the missense variants focusing on the amino acid and not on the nucleotide variant itself, the exonic mutation causing splicing alterations are often misclassified as synonymous, missense, or nonsense variants. Thus, it is possible that their effect on gene expression, including pre-mRNA processing, may be overlooked. However, as discussed below, this possibility should not be neglected, since several reports have disclosed the effects of missense DNA variants on mRNA, as reviewed in [28].

### 3.3. Deep Intronic Variants

Deep intronic variants are localized within large introns, far from exon boundaries. Such variants may generate novel acceptor or donor sites, which are bound by the spliceosome complex and used in combination with the existing intronic cryptic splice sites. They may also create novel regulatory elements and lead to the recognition of the specific intronic sequences as exonic sequences (detailed review in [32]). As a result, such variants may lead to the inclusion of an intron fragment, called pseudo-exon, into the mature transcript. The inclusion of a pseudo-exon in the mRNA generally modify the reading frame introducing a premature stop codon [33].

Deep intronic mutations are not common, and difficult to identify since are located in regions not usually analyzed in routine procedures. However, it has been becoming increasingly evident that deep intron regions play an important role in different physiological and pathological mechanisms related to mRNA processing [34,35]. Since the effect of intronic variants on transcript splicing and protein synthesis may be significant, the analysis for their presence should be considered when the standard screening of coding regions and exon/intron boundaries is not conclusive.

## 4. Identification of Splice Variants in NGS Era

An accurate classification of genomic variants is the cornerstone of genomic and precision medicine. Only identifying the causative variant of inherited disorders and evaluating its actual consequences on proteins and cells is possible to offer a helpful genetic counseling and improve patients’ clinical management. The recent advent of next generation sequencing (NGS) technologies has allowed obtaining an accurate identification of the variants present in an individual’s genome, revolutionizing the times and ways to achieve genomic data. Gene panels, exome and genome sequencing consent to identify the majority of coding variants for several disorders [36]. However, despite these huge technical improvements, the biological and clinical interpretation of a large part of identified variants remains challenging [37]. This difficulty is particularly evident in the identification of splice variants.

It has been estimated that up to 15% of all point variants causing human genetic disorders involve splice site consensus sequences, particularly at intronic positions, resulting in splicing defects [38]. The percentage of splicing variants reported in the Human Gene Mutation Database (HGMD) is about 9% (27,959/323,661) (HGMD database, accessed on 5 August 2021). However, this number seems underestimated, since it only marginally takes into account nucleotide substitutions in coding regions, which are usually considered as missense, nonsense, or silent variants. Based on in silico data, it has been reported that the proportion of exonic variants that may affect splicing, but have been originally classified as missense/nonsense in the HGMD, can reach up to 25% of all the variants present in the database [39,40]. In addition, not only point variants but also other genetic variants, such as small indels, can modify cis splicing regulatory elements and affect the splicing process [41]. These data indicate that variants affecting splicing play an important role in the etiology of genetic disease and underline the importance of a correct variant interpretation.

The characterization of potential splice variants is usually based on the analysis of RNA from the patient or some other laboratory techniques, including in vitro assay [38]. However, laboratory tests for splicing variants are expensive and time-consuming, so other approaches have been set up to reduce costs and times of analysis. The use of in silico prediction tools allows focusing on those variants with real chance of being deleterious and selecting them for further experimental validation.

## 5. Predictive Tools for Splice Variant Identification

The tools available for splicing analysis were originally developed for research purposes; however, they have been becoming integral part of the diagnostic process, as a first step of variant characterization. In general, splice site prediction tools have increased sensitivity (~90–100%) relative to specificity (~60–80%) in predicting splice site abnormalities [4].

Several algorithms have been proposed, differing among each other in the approach they use for splice variant prediction. They can be divided into two big categories: early computational methodologies and the more recent Machine Learning-based tools.

### 5.1. Early Computational Methodologies for Splice Variant Prediction

The main differences among these methodologies rely on the consensus sequences they used for the comparison with the input sequences, and the statistical model used for the analysis. Table 2 shows the key features of some of these tools.

#### 5.1.1. Input Sequences

Most of the tools focus on the analysis of consensus splicing donor and acceptor sequences at exon-intron junctions and require the sequence input at least including positions from −3 to +6 in the case of 5′ donor site and or from −20 to +3 for the 3′ acceptor sites. Examples of these tools, based on different computational models, are SpliceView [42], GeneSplicer [43], Spliceport [44], GENSCAN [45], NetGene2 [46], NNSplice [47], and MaxEntScan [48].

Other tools have been developed to predict whether a single nucleotide variant can affect the branch site motif or polypirymidine tract, e.g., SVM-BPfinder [49] and IntSplice [50].

A more limited number of algorithms analyze the input sequence to predict exon skipping, cryptic site activation, or generation of aberrant transcripts (CRYP-SKIP [51]), or to identify though and how distant a variant may influence the splicing process (Spliceman [52]).

Several tools have been built to predict the effect of a specific variant on exonic splicing enhancers (ESEs) and exonic splicing silencers (ESSs). These tools may be very useful in the characterization of exonic variants. Examples of this kind of algorithms are ESE Finder [53,54], ESRseq [55], and FAS-ESS [56], all three based on individual experimental data, HEXplorer [57], and RESCUE-ESE [58], which rely on computational analysis of nucleotide motifs or k-mer distributions, and SpliceAid [59], searching for interactions between validated RNA target motifs and human splice regulatory proteins.

Other tools focus on motifs involved in the binding to RNA-binding proteins (RBPs). RBPmap uses motifs well characterized in the literature and analyzes their evolutionary conservation to define potential binding sites [60]. Splicing Factor Finder performs a mapping of splicing factor binding sites considering both genomic environment and evolutionary conservation of the regulatory motifs [61].

Finally, other bioinformatic tools perform predictive analysis evaluating whether a variant may affect mRNA secondary structure. Examples of these algorithms are pFold or UNAFold [62,63].

#### 5.1.2. Statistical Models

One of the most frequently used algorithm is the basic Position Weight Matrix (PWM) model [64], which scores and ranks each nucleotide on the splice site sequence based on its frequency from its aligned consensus sequence. The PWM model has been used in several tools, for example in the SpliceView [42], which considers mutual dependency between nucleotides in different positions.

The Maximal Dependence Decomposition (MDD) model, used in GENSCAN [45], is a decision tree method that captures most significant dependencies between positions by dividing the dataset into subgroups and modeling each subset separately. The MDD model has been implemented by adding Markov models (MM), which identifies additional dependencies among adjacent positions, in the tool GeneSplicer [43].

The Maximum Entropy Distribution (MED) is probably the method that currently allows the most unbiased approximation for modeling short sequence motifs. MED can be considered as a framework, rather than a single model, which enables to generate different models by modifying the applied constraints. MED only assumes that the distribution is consistent with the empirical features which are obtained from known data. It takes into account dependencies between both adjacent and non-adjacent positions. The tool MaxEntScan [48] uses this approach and shows high flexibility, since the user may choose between default or personalized models. In addition, MaxEntScan can employ other algorithms, such as the PWM, MDD, and MM models, to perform the analysis and compare the results.

#### 5.1.3. Tools Combining Multiple Algorithms

Some tools utilized different algorithms to implement the strength of the analysis. Human Splicing Finder [65] performs predictions using the PWM and MED models and analyzing branch points, ESEs, and ESSs. SROOGLE is a webserver based on nine different algorithms able to analyze sequences belonging to thirteen groups of splicing-regulatory sequences [66]. Automatic Analysis of SNP sites (AASsites) employs five gene prediction programs to evaluate the impact of SNPs on splicing [67]. Finally, EX-SKIP and HOT-SKIP examine the probability that substitutions in each exonic position cause exon skipping, using several integrate approaches to analyze potential ESE/ESS sequences [68].

**Table 2:** List of predictive tools and used strategies.

Tool Name	Analyzed Regions	Predictive Model	URL	Ref.
**Canonical Splice Sites**				
MaxEntScan	5′ and 3′ SSMs	PWM, MDD, MM, and MED	http://hollywood.mit.edu/burgelab/maxent/Xmaxentscan_scoreseq.html (accessed on 3 September 2021)	[48]
SpliceView	5′ and 3′ SSMs	PWM	http://bioinfo.itb.cnr.it/~webgene/wwwspliceview.html (accessed on 3 September 2021)	[42]
GeneSplicer	5′ and 3′ SSMs	MDD	https://www.cbcb.umd.edu/software/GeneSplicer/gene_spl.shtml (accessed on 3 September 2021)	[43]
Spliceport	5′ and 3′ SSMs	SVM	http://spliceport.cbcb.umd.edu/ (accessed on 3 September 2021)	[44]
GENSCAN	5′ and 3′ SSMs	MDD	http://hollywood.mit.edu/GENSCAN.html (accessed on 3 September 2021)	[45]
NetGene2	5′ and 3′ SSMs	NN	http://www.cbs.dtu.dk/services/NetGene2/(accessed on 3 September 2021)	[46]
NNSplice	5′ and 3′ SSMs	NN	https://www.fruitfly.org/seq_tools/splice.html (accessed on 3 September 2021)	[47]
SVM-BP Finder	BPs + PPT	SVM	http://regulatorygenomics.upf.edu/Software/SVM_BP/ (accessed on 3 September 2021)	[49]
IntSplice	BPs + PPT	SVM	https://www.med.nagoya-u.ac.jp/neurogenetics/IntSplice_v1.0/index.php (accessed on 3 September 2021)	[50]
**Cryptic sites**				
CRYP-SKIP	exons + flanking intronic sequences	multiple logistic regression	https://cryp-skip.img.cas.cz/ (accessed on 3 September 2021)	[51]
Spliceman	variant + flanking nucleotides	L1 distance metric	http://fairbrother.biomed.brown.edu/spliceman/ (accessed on 3 September 2021)	[52]
**Exonic Sequences**				
ESE Finder	ESE	PWM	http://krainer01.cshl.edu/cgi-bin/tools/ESE3/esefinder.cgi?process=home (accessed on 3 September 2021)	[53,54]
RESCUE-ESE	SREs	experimental + computational approach	http://hollywood.mit.edu/burgelab/rescue-ese/ (accessed on 3 September 2021)	[58]
ESRseq	ESE + ESS	PWM	https://www.ncbi.nlm.nih.gov/pmc/articles/PMC3149502/ (accessed on 3 September 2021)	[55]
Hexplorer	SREs	experimental + computational approach	https://www2.hhu.de/rna/html/hexplorer_score.php (accessed on 3 September 2021)	[57]
FAS-ESS	ESS	MED	http://hollywood.mit.edu/fas-ess/(accessed on 3 September 2021)	[56]
SpliceAid	ESE + ESS + ISE + ISS	scanning against validated splicing sequences	http://www.introni.it/splicing.html (accessed on 3 September 2021)	[59]
**Conservation**				
RBPmap	RBP sites	Weighted-Rank (WR)	http://rbpmap.technion.ac.il/ (accessed on 3 September 2021)	[60]
Splicing Factor Finder	RBP sites	WR	https://pubmed.ncbi.nlm.nih.gov/19296853/ (accessed on 3 September 2021)	[61]
**RNA Secondary Structure**				
pFold	RNA sequence	stochastic context-free grammar (SCFG)	https://pubmed.ncbi.nlm.nih.gov/12824339/ (accessed on 3 September 2021)	[62]
UNAFold	RNA sequence	free energy minimization, partition function calculations, and stochastic sampling	http://www.unafold.org/ (accessed on 3 September 2021)	[63]
**Combined Analysis**				
EX-SKIP	ESEs + ESSs	four algorithms	https://ex-skip.img.cas.cz/ (accessed on 3 September 2021)	[68]
HOT-SKIP	ESEs + ESSs	four algorithms	https://hot-skip.img.cas.cz/ (accessed on 3 September 2021)	[68]
Sroogle	SSM + BP + PPT + SRE	nine algorithms	http://sroogle.tau.ac.il/ (accessed on 3 September 2021)	[66]
Human Splicing Finder (*)	SREs, splice sites or branch sites	PWM and MED	http://www.umd.be/HSF3/ (accessed on 3 September 2021)	[65]

(*) free only for academic use. SSMs: Splice Site Motifs; BPs: Branch Site Motifs; PPT: PolyPirymidine Tract; ESE: Exonic Splicing Enhancer; ESS: Exonic Intronic Splicing silencer; ISE: Intronic Splicing Enhancer ISS: Intronic Splicing Silencer; SRE: Splicing Regulatory Element; RBP: RNA Binding Protein; ORF: Open Reading Frame; PWM: Position Weight Matrix; MDD: Maximal Dependence Decomposition; MM: Markov models; MED: Maximum Entropy Distribution; SVM: Support Vector Machine; NN: Neural Network.

### 5.2. Machine Learning-Based Tools

The name “Machine Learning” (ML) was used for the first time in 1959 by Arthur Samuel, who defined ML as the “field of study that gives computers the ability to learn without being explicitly programmed” [69]. ML methods generally analyze previously collected data to build data-based models, find out statistically significant patterns, and on this basis make predictions on novel data. Therefore, it can be said that ML algorithms are able to “learn” from datasets and utilize the acquired knowledge to analyze similar data [70].

Algorithm “training” is usually performed using experimentally verified pathogenic variants as positive examples, and known benign variants as negative reference. In this way, ML software progressively identifies patterns able to discriminate between pathogenic and benign variants and subsequently uses these patterns to correctly predict whether a new variant may be pathogenic or not. During the training, some specific algorithms are used to develop an initial model. The model is then challenged on a test set and its efficacy is evaluated. In this way, the model can be progressively improved to maximize its efficacy.

Some elements are fundamental in the learning process. First, all ML models need both training and testing datasets, which must be absolutely independent from each other. In other words, if an entry is present in one set, it should not appear in the other one. To obtain this, a dataset is often divided into two subsets that are used as training set and test set, respectively. The lack of overlapping ensures better results, since it avoids that the model recognizes in the test set the same items it had already seen in the training phase, and therefore displays a performance better than real [36]. Moreover, it is important to balance positive or negative datasets, as the excess of positive datasets can cause underfitting and that of negative dataset can generate overfitting models [36].

The variables in a dataset that are input to a ML model are called “features”. Data are classified or separated based on these variables. Different features may be used: many of them are often sequence-based, e.g., the frequency or position of specific nucleotides in a given region, others are biochemical features, such as GC content and thermodynamic properties.

The availability of public datasets of variants is very important for developing ML-based prediction tools. Among these databases, an important role is played by experimentally-derived RNA-seq datasets, which provide an effective link between genome and transcriptome features, and databases that report a classification variants based on potential pathogenicity, such as ClinVar [71].

Regression and classification algorithms are used for prediction in Machine learning. Regression algorithms are used to make prediction on continuous values, while classification algorithms are used on discrete data. They divide the data into different classes and are used to identify the class to which a new data entry is most likely to belong. Table 3 reports a brief description of the different methods used in machine learning, exhaustively reviewed elsewhere [72,73].

Several ML-based prediction algorithms have been developed in the last years. They mainly differ in ML architecture, experimental datasets they use, and functions they propose. The main ML tools used for splice prediction are shown in Table 4, including details about ML methods, training/testing datasets, strengths and weaknesses of each tool.

Among the earliest ML-based tools, CADD [74,75] has been trained using both benign and pathogenic variant sets. It outputs a score that can be interpreted as a measure of pathogenicity. The first version of CADD used an SVM-based approach. Subsequently, L2-regularized logistic regression—a kind of regression model allowing the modeling and prediction of a binary dependent variable—has been adopted since it leads to improved sensitivity and specificity [74]. The CADD scoring has soon become a gold standard for the prediction of variant impact and the reference to evaluate other predictive tools. However, it has some limits that may weaken its efficacy: it uses conservation scores, thus it is really effective for protein-coding impact prediction, but it is lacking in predicting variant effect at the transcript level [36].

This limit is overcome by TraP [76], a random forest-based tool, which analyzes non-coding variant impact at the transcript level, providing a score between 0–1. The score can be used as a measure of the impact a variant is likely to have on a transcript. It has been shown that TraP scoring works better than the CADD model on the prioritization of variants impacting on splicing. In addition, TraP identifies also pathogenic intronic variants and evaluates the potential impact of variants across multiple transcripts, a feature usually not considered by many prediction tools [77].

Another tool is CryptSplice [78], an SVM-based method, which aims to predict the variant impact on generation of cryptic splice sites. It evaluates three situations: a canonical site weakened by the introduction of a new splice site in its proximity, a canonical site replaced by a novel site, and the introduction of a functional deep intronic splice site.

S-CAP [79] is an example of a tool designed to predict the pathogenicity of splice-impacting variants. S-CAP distinguishes and separately analyzes 6 distinct regions,: 3′ intronic, 3′ canonical site, exonic, 5′ canonical site, 5′ extended, and 5′ intronic, all within 50 bases from the canonical exon-intron junction. This approach tries to overcome the limit of most ML models that tend to prioritize canonical splice site variants and underestimate the pathogenicity of intronic variants.

Another approach has been used to develop the tool PEPSI (Prediction of variant Effect on Percent Spliced In) [80], a random forest regression model trained on multiple layers of features related to sequence conservation and regulatory sequence elements. Its peculiarity is to integrate secondary structure information in predicting variants that disrupt splicing regulatory elements (SREs). In a comparative analysis with other splice prediction tools, PEPSI framework has shown comparable sensitivity and precision in predicting variants able to alter splicing. Nevertheless, the approach of PEPSI of evaluating SRE changes based on the probability of secondary structure formation has displayed several limitations that may reduce its effectiveness in detecting splice-disrupting variants.

SpliceAI [81], a deep learning tool consisting of a 32-layer deep residual neural network, analyzes each position of a pre-mRNA transcript and assesses the probabilities it is a splice donor, splice acceptor, or neither. SpliceAI has been designed to infer features from the transcript sequence itself. It generates scores for gain or loss of acceptor or donor for all nucleotides within 50 bp of the variant of interest. Then, for each of these four possibilities, the nucleotide within the region affected by the most significant change is returned. When used in a near-agnostic approach to model training, SpliceAI is able to identify novel features by itself, potentially increasing global knowledge of splicing process. Considering the power of the model, SpliceAI may be considered the current gold standard for clinical interpretation of splice-impacting variants.

**Table 4:** List of ML prediction tools with the kinds of used strategies.

Tool Name	Prediction	Model	Datasets	Key-Points	Ref
CADD	Score of pathogenicity	Rirst version: linear SVM Later versions: L2-regularized logistic regression	Training datasets: Benign: evolutionarily neutral variants; Pathogenic: simulated de novo pathogenic variants Testing datasets: Benign: benign variants; Pathogenic: pathogenic ClinVar variants, somatic cancer mutation frequencies	Effective tool for protein-coding impact prediction; may not be informative for poorly-conserved regions	[74,75]
CryptSplice	Impact of variants on existing splice sites, cryptic splice site prediction	SVM with RBF kernel	True and false splice sites from GenBank-derived datasets	Identify creation of cryprtic acceptor/donor site; use of a quite obsolete database	[78]
DARTS	Prediction of alternative splicing using both cis sequence features and mRNA levels of trans RBPs	DNN and Bayesian Hypothesis Testing	RNA-seq data (*)	Evaluation of RBP impact on splicing	[82]
MMSplice	Multiple predictions: exon skipping, competitive interactions, changes in splicing effciency, and pathogenicity	Modular NN, linear and logistic regression	Donor/acceptor modules: GENCODE v24 true (known sites) and false (random sequences) splice sites Exon/intron modules: MPRA data from [83]	Easily clinically applicable training set; contains false positive/unverified sites	[84]
MutPred Splice	Impact of coding region substitutions on disruption of pre-mRNA splicing	Linear SVM	Positive: HGMD exonic disease-causing/disease-associated variants Negative: HGMD disease-causing missense, not reported to disrupt exon splicing, high frequency exonic SNPs (SNP- from 1000 Genomes Project [85]	Suitable for use in an NGS high-throughput setting to identify and prioritize potentially splice-altering variants	[86]
PEPSI	Prediction of coding and noncoding variant impact on pre-mRNA splicing based on sequence conservation, RNA secondary structure, and regulatory sequence elements	Random forest regression model	Data obtained form the Vex-seq experiment (measurement of the ΔPSI of 2055 variants from the Exome Aggregation Consortium (ExAC; [Kircher et al., 2014]) v24 a selection of chromosomes as training set, the remaining ones as testing set (*)	Indels and intronic variants included	[80]
S-CAP	Score of variant pathogenicity using compartmentalization of genomic regions	DNN	Pathogenic variants selected from HGMD and ClinVar; benign variants from gnomAD	Evaluation of intronic pathogenic variants; variants lying more than 50 bp into the intron are not covered by the model	[79]
SPANR	Cassette exon skipping prediction	NN modeled on Bayesian framework	PSI values for all human exons across 16 tissues, based on the Illumina Human Body Map project (*)	Web server easy to use, availability of a dataset of pre-computed scores for all eligible variants in the genome; evaluation of exon sequence only	[87]
SpliceAI	Prediction of variant impact on loss or gain of acceptor/donor sites	32-layer DNN	Protein-coding transcripts from GENCODE v24 (a selection of chromosomes as training set, the remaining ones as testing set) (*)	Very powerful tool able to use a “near-agnostic” approach	[81]
SpliceFinder	Classification of variants based on impact on donor site, acceptor site or non-splice-site	CNN	Sequences of donor, acceptor, and non-splice-site, randomly selected from human reference genome (90% for training, 10% for testing, and then 20% of the training data for validation)	Non-canonical splice sites can also be predicted correctly; decreased number of false positives	[88]
TraP	Quantification of impact of variant on transcripts	Random forest	Benign: De novo mutations in healthy individuals Pathogenic: selected synonymous variants associated with rare disease (*)	High performance in distinguishing pathogenic and benign variants, both intronic and synonymous; evaluation of potential impact of variants across multiple transcripts	[76]

(*) data from NGS experiments. SVM: Support Vector Machine; RBF: Radial Basis Function, DNN: Deep Neural Network; NN: Neural Network; CNN: Convolutional Neural Network.

## 6. Interpretation and Evaluation

Considering the number of in silico tools available for splice variant impact prediction, the choice and interpretation of results may be challenging. It may sound obvious, but the starting point for a good result analysis is to know the bases and the assumptions of the different tools.

A tool predicting competitive splice site interactions, for example, gives information different form one predicting exon skipping, and their results can be conflicting, simply because they analyze diverse features. On the other hand, this can become a strength for the prediction, since the different approaches adopted by the different tools provide the users with the possibility to evaluate variant impact from many perspectives. In general terms, in silico tools may perform predictions either on splicing impact or pathogenicity of a variant. In the first case, most tools report analysis results as a score, that is a numerical measure of the strength of the splicing signal. The range may varies, but in general a higher score corresponds to a stronger similarity to the consensus sequence or a greater probability that a site is a true splice site. However, a score is just a number whether there is no an affordable threshold separating positive sites from negative ones. It is possible to set a cutoff value to evaluate though a variant is causing splicing defects, but this value is usually arbitrarily chosen by the users and can change across different tools in different studies [89]. Therefore, it may provide useful information, but should not be regarded as an absolute reference to discriminate between variants.

In the case of tools predicting variant pathogenicity, users should be aware that the training of a model is based on human annotations of pathogenicity, reported in databases as ClinVar [71]. These annotations reflect the current variant classification and the current knowledge of splice-impacting variants, and probably report some misclassification for the less characterized splice variant types [36]. This is a common bias of prediction tools: all of them are based on, or learn from, available experimental data and databases, thus they can be improved only obtaining a higher number of validated data. For this reason, a continuous update of databases is fundamental to progressively implement and refine prediction reliability.

Based on these considerations, as a general approach, the use of multiple tools, relying on different assumptions, for splice variant impact prediction is recommended. The different programs have different strengths and weaknesses, depending on the algorithm they use, and this may allow reducing the possibility of errors. Of course, since the practical use of tools and the result interpretation is not always easy and often time-consuming, the tools that analyze more features simultaneously may be very helpful.

On the other hand, care may need to be taken with the tool selection: many of them do use different algorithms, but these algorithms are actually based on similar assumptions. In this case, the combination of predictions from different tools does not strengthen the analysis and should be considered as a single evidence in variant interpretation [85]. In addition, many tools share common limits. Only few tools (CADD, MMSplice, and SpliceAI), for example, are able to predict the splice impact of indels, even though indels involving specific region, as the PPT, may exert relevant effects on splicing even more than single nucleotide variants [90,91]. Additionally, deep intron variants are rarely included in the analyzed regions or in the training sets, thus many tools may be poorly effective in predicting splice modifications involving these low-frequency sites.

Another underestimated mechanism is the presence of long-distance splicing interactions: splicing may be also affected by the interactions of trans-acting splicing complexes with binding sites across all intron lengths [92,93]. SpliceAI considers a wider genomic context than other tools, with a significant increase of model performance. In addition, this tool may be very useful in the research of long-range determinants of splicing, providing novel information and eventually increasing and deepening our knowledge of splicing mechanisms.

As better discussed below, the ACMG guidelines have recently defined the criteria for splicing variant evaluation [4]. In particular, they state that the computational evidence should not be overestimated, also considering that the algorithms can have vastly different predictive capabilities for different genes. In general, only though all the predictive tools agree on the prediction, this evidence can be counted as supporting. However, these are anyway predictions, and their use in sequence variant interpretation should be cautious. It is not recommended that they be used as the only source of evidence for clinical and diagnostic aims, but any positive findings from in silico tools necessitate to be confirmed using in vitro approaches.

## 7. Validation of Predicted Splicing Variants

Validation methods, which complement and substantiate predictive analyses, consist in the studies of the functional effect produced by a potential splicing variant. Functional testing can be performed on RNA (transcriptomics analysis) and/or at protein level (proteomics experiments) [26,27].

### 7.1. Transcriptomics Functional Testing

Transcriptome analysis focuses only in the protein-coding region of a gene, facilitating the detection of variants that influence RNA expression rather than detection on DNA [94]. Before the description of different functional testing, the major advantages and disadvantages of RNA handling need to be explained. Although RNA isolation from patients is considered a simple and fast procedure, RNA manipulation is not so easy. Other weaknesses are represented by the purity and the degradation rate of this genetic material. In practice, the identification of cell lines and/or tissues as optimal source of RNA is still challenging. In the majority of cases, blood (leukocytes) or cultured cells (generally fibroblasts) represent the best options to isolate a huge amount of RNA from patients in order to identify splicing defects [94,95]. Tissues may be the ideal source for comparison of effects resulting from aberrant splicing in healthy and affected samples and should definitively determine if the splicing mutation causes disease. However, the appropriate tissues are often not available and, when available, the genetic material suffers from fixation treatment, so it is hard to obtain high yield of RNA [95,96]. In addition, RNA is a highly prone-to-degradation molecule and the NMD process [97] represents the predominant cause of false-negative results in RNA analysis. If cells tend to prevent the translation of aberrant splice transcripts (carrying the mutated allele), which are commonly degraded, only the normal allele is detectable (in heterozygosis condition) and splicing cannot be proved [29,94]. In order to circumvent NMD, patients’ cells need to be treated with puromycin or cylcoheximide (the most common NMD inhibitors) to stabilize RNA and resolve this intrinsic problem [26,96,98].

Experimental procedures for identification of the alternative splicing sites can be classified into two groups on basis of their degree of multiplexity, which is a measure of how many different genes can be investigated by a given experiment. The class of “low to mid-plex methods” includes Northern blotting, RT-PCR and minigene assay, while microarrays, Tiling array and RNA-seq are methods belonging to the “higherplex technologies” [99].

Northern blotting is a relatively old technique that can be performed for detection and quantitation of mRNAs in order to determine whether the predicted variant affects splicing. The procedure is based on hybridization of patient-isolated RNA with specific radioactively-labeled RNA probes to obtain information about size and amount of RNA encoded by the gene of interest [100]. Quantifying RNA is useful to measure the expression of a particular gene, and this method can also provide a direct comparison of RNA level between several samples, based on size disparity between differentially spliced transcripts by electrophoresis [101]. In general, Northern blot requires a huge amount of RNA and measures only steady-state mRNA levels. All these limitations lead to choose the PCR, a more accurate technique, as preferred validation method of predicted splicing variants [102].

Reverse transcription PCR (RT-PCR) is one of the most used and low-cost methods to reveal if the identified variant can influence the mRNA sequence [26,27]. This highly sensitive approach, consisting in the amplification of the target sequence and following detection of products on agarose gel, requires a low quantity of RNA for the analysis of a large number of samples and several different genes in one single experiment. Over the years, a multitude of PCR-based strategies has been developed, followed by Sanger sequencing, to successfully identify the precise mutation causing aberrant splicing.

An alternative method to RT-PCR and sequencing is represented by the minigene assay, which compares the splicing mechanism of mutant and wild-type exons within an alternatively spliced gene [103]. It is based on the cloning of the specific sequence of interest, with and without mutations, in a plasmid. In case of exonic mutations suspected of affecting splicing, the exon and a small amount of flanking intronic regions are inserted into the construct, whereas deep intronic mutations can be detected inserting into the minigene the two exons surrounding the intronic region of interest. Cells transfected with the plasmid will produce the mRNA derived from the minigene that can be selectively amplified by RT-PCR and then analyzed on agarose gel [96]. This system may be useful for analysis of genes with a reduced expression in leukocytes or fibroblasts [104].

Several advantages over previous approaches have been obtained with the development of high-throughput technologies, either hybridization- or sequence-based, to unravel the complexity of transcriptome. In particular, Microarrays and direct RNA sequencing have been widely used in order to validate the in silico predictions [29,105].

**Figure 3:**
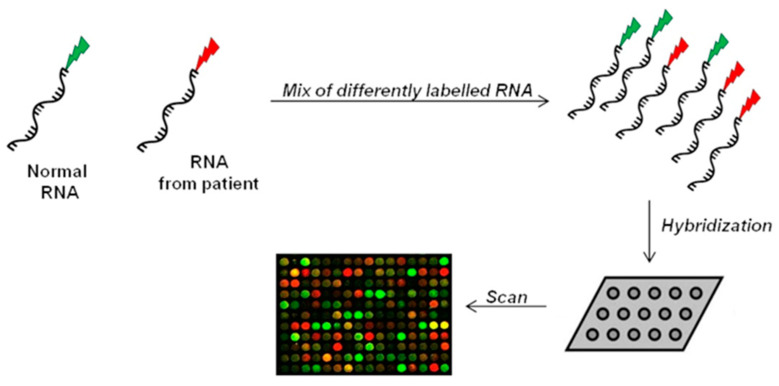
Microarray technology. RNA of two samples (normal/reference RNA and patient-isolated RNA) are differently labeled, mixed, and then spotted on the same microchip. After hybridization, the chip is scanned at two wavelengths to capture signals of the two different dyes. Scanner of the array generates an image for interpretation of the results. Green spots indicate expression in normal cells, while red spots indicate only expression in affected cells. Yellow signal means co-expression (not significant result).

The microarray method belongs to the hybridization-based approaches. It uses microchips covered with short probes for the large-scale analysis of gene expression [106]. Patients’ isolated-RNA and reference RNA are fluorescently labelled and then hybridized on the array. Following hybridization, fluorescent signals on microarray are captured by a laser system, generating an image to evaluate gene expression and for subsequent data processing (Figure 3) [107,108].

Monitoring simultaneously thousands of genes, microarray approach can detect splice site mutations and identify diagnostic or prognostic biomarkers which allow to discover a different expression pattern in healthy and disease conditions [109]. However, the sensitivity of microarray (detection range comprised between 1 and 10 copies of mRNA per cell) may result insufficient in case of low-expressed genes, limiting detection of relevant changes [108,110]. The whole-genome tiling array, an updated version of microarray, has been designed to cover the entire genome and not only specific regions, providing a global and more unbiased view of gene expression in samples with different clinical conditions [111,112]. Nevertheless, both conventional and whole-genome microarrays are affected by numerous sources of noise, such as background problems and non-specific hybridization [113,114], threatening the reliability of analysis [105].

Recent advances in new sequencing technologies have triggered an increasing shift from hybridization arrays towards sequence-based methods, in order to improve the detection of novel splicing sites [115,116]. For example, RNA-seq (RNA sequencing) has emerged as a new tool for the investigation of the whole transcriptome by directly sequencing cDNAs, improving gene expression studies, and unraveling the complex nature of alternative splicing mechanism [116]. As reported by Saedian and collaborators, the power of RNA-seq technology resides in the capability to identify pathogenic variants which cannot be captured by Whole Exome Sequencing (synonymous/silent and nonsynonymous/nonsense exon variants or mutations occurring in deeply intronic regions) probably affecting splicing events [26,117,118].

**Figure 4:**
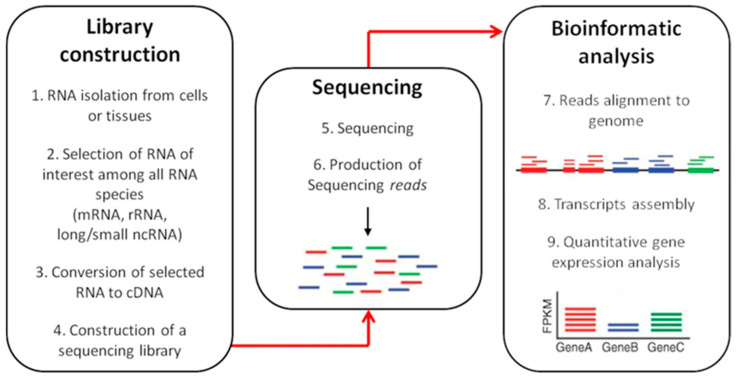
RNA-sequencing workflow. RNA-seq is a three-step method: (1) library construction; (2) sequencing; (3) bioinformatic analysis. The RNA species of interest is selected and converted to complementary DNA, which is then amplified by PCR in order to prepare a sequencing library. Sequencing results in the generation of short sequences (reads) that need to be aligned to a reference genome. Then, different approaches can be used for transcript assembly to detect quantitative gene expression.

RNA-seq workflow is depicted in Figure 4.

Briefly, the initial phase consists in the RNA isolation using standard procedure, followed by the selection of an RNA subtype among different subpopulations (mRNA, tRNA, ncRNA, miRNA) [116]. The construction of an appropriate RNA-seq library is the next key step, which determine how accurately the final sequencing output reflects the original transcriptome [105]. RNA is fragmented to create short transcripts (200–500 bp) in order to minimize secondary structure formation and to reduce end biases [119]. RNA sequences are then converted into cDNA which undergoes 3′-adenylation and ligation of adaptor molecules to both ends of the fragments before amplification through PCR [120]. PCR products are then subjected to sequencing that will produce shorts sequences (reads) to align with a reference genome to perform the gene expression profile [121].

RNA-seq provides a powerful tool for transcriptome-based applications beyond the limitations of microarrays, but it also has some pitfalls. Benefits and drawbacks of the two methodologies and the main differences between them are following discussed and listed in Table 5.

First of all, RNA-seq analysis consists in the full sequencing of the whole transcriptome and can detect a larger percentage of differentially expressed genes compared to microarrays which are limited to pre-defined genes and analyze only a portion of protein-coding regions. Together with the higher specificity and sensitivity, an important benefit of RNA-Seq over microarrays is represented by its ability to quantify almost all types of RNAs, mapping the whole genome and enabling identification of new transcripts and previously unrecognized splice variants. By contrast, microarray requires the indispensable a priori knowledge of the sequences being investigated and transcript-specific probes [116], which reduce gene expression analysis across a narrower dynamic range, significantly limiting new splicing variants discovery [121,122,123].

However, the RNA-Seq approach has some challenges that prevent a complete technological switch to sequencing in gene expression profiling: (1) RNA-seq produces large size files, which are considerably more complex than microarray results; (2) Sequencing data analysis requires an advanced bionformatic approach and expensive computational tools; (3) There are no standard protocols and adequate reference databases, which make data interpretation more difficult; 4) although RNA-seq has become increasingly affordable, RT-PCR followed by Sanger sequencing is more manageable in term of costs [121].

### 7.2. Proteomics Analysis

**Figure 5:**
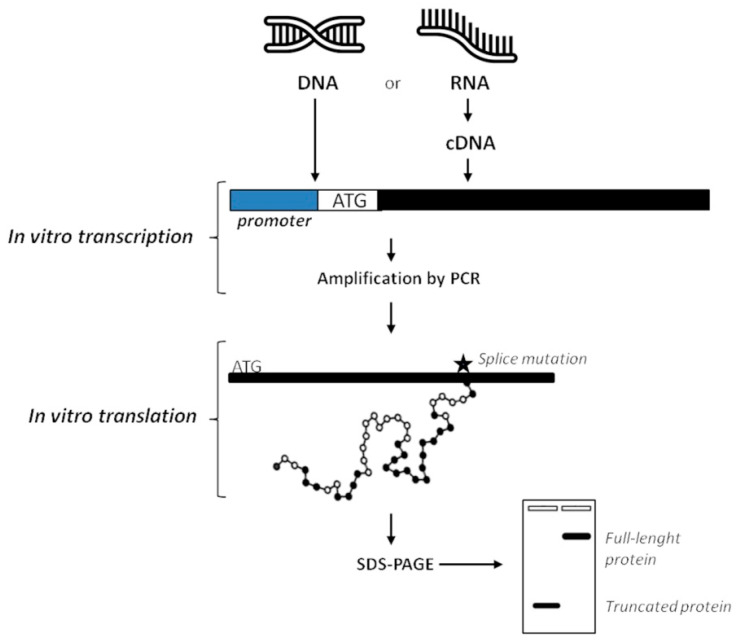
Overview of the protein truncation test. DNA or cDNA obtained from RNA by retrotrascription can be used as a template to perform PCR. During amplification process, an RNA polymerase promoter and a translation initiation sequence (ATG) are added to products, together with a consensus Kozak sequence to improve the process. Then, the RNA polymerase promoter initiates transcription and the ATG sequence is used to start translation of RNA into protein. PCR fragments are then separated on basis of their size by agarose gel-electrophoresis, and mutations affecting splicing can be revealed. In the radioactive PTT, addition of radiolabelled amino acids in nascent proteins requires blotting after SDS-PAGE and then exposition to X-ray to analyze results (not shown). Finally, only DNA sequencing can confirm if the production of truncated proteins is due to aberrant splicing.

Differently from functional testing on genetic materials, proteomics analysis is usually performed by immunohistochemistry. By contrast to RNA-based techniques, proteins are not commonly isolated from patients’ samples because of high risk of contamination during the extraction procedure that can mostly give low yields of product [94,95]. In order to test a protein on a functional level, the Protein Truncation Test (PTT) or In-Vitro Synthesized Protein assay (IVSP) [124] was developed to identify variants that introduce a premature stop codon, compromising protein translation. In practice, the procedure consists of a RT-PCR followed by in vitro translation of the PCR product into proteins or radiolabelled proteins through the 3H-Leucine incorporation. Performing the SDS-PAGE, proteins are separated on basis of their size. Additionally, when radioactive amino acids are used, gel is then blotted and exposed to X-ray. In both non-radioactive and radioactive PTT the analysis will reveal if shorter than normal-size variants are synthesized. Obtaining proteins of lower mass than the expected full-length proteins means that there are mutations in the analyzed gene (i.e., deletions, duplications, and variants affecting splicing) affecting the normal RNA processing (Figure 5) [26,125].

Once truncated proteins have been identified, an in vitro assay could then be designed to directly test their function in cellular pathways and biological processes, for example, their DNA binding properties or enzymatic activity. Of course, performing DNA sequencing, splicing site mutations can be validated as variants encoding aberrant proteins.

Of note, false positive PTT results only rarely occurs, by contrast of several causative events that might produce false negatives results: low-purity RNA and errors during amplification process [94].

Several improvements have been made over time to the original procedure in order to increase the experimental throughput: in example, the substitution of radioactive-labeled with biotin-labeled amino acids has facilitated detection through fluorescent-conjugated antibodies, or the use of specific protein tagging N- and C-terminal sequence of the synthesized proteins has allowed to detect truncating changes without performing SDS-PAGE [125,126].

Two-dimensional gel electrophoresis, Western blotting, and mass spectrometry are considered alternative methods to the PTT assay, even if they detect truncating variants as well as variants carrying amino acid substitutions [94,111], without testing functional activity of the mutated protein.

Despite advancements in the procedure and the employment of alternative methods, PTT has been mostly replaced by sequencing technology; however, it still remains a good method to test functional activity of aberrant proteins already validated by transcriptomics, with a detection efficiency close to 100% [26,94].

## 8. Splice Variant Characterization in Diagnostics

The recent NGS technologies allow sequencing large panel of genes, or whole exomes and genomes, for a wide range of disorders, and identifying candidate causative variants for these conditions. The assessment of the real functional impact of variants on genetic diseases is a key element in the proper interpretation of their clinical significance. This evaluation may be particularly challenging in the case of variants affecting the splicing process. While the variants that impact donor and acceptor splice site motifs are usually identified as splice variants, exonic and intronic variants outside of the donor and acceptor splice site motifs are often overlooked. The American College of Medical Genetics and Genomics (ACMG) have recently developed updated guide lines for the interpretation of sequence variants, including splice site variants [4]. ACMG guidelines remind that it is important to evaluate the possibility that a variant may act directly through the specific DNA change rather than through the amino acid change. Exonic variants should not be annotated as synonymous, missense, or nonsense, based on predicted codon and the amino acid they affect, but an analysis of their impact on splicing should be performed. Of course, this analysis should take into account the patient’s clinical history. For example, the segregation of the variant with a phenotype in a family is evidence for the association of the variant with the disorder, even though that variant has been classified as “silent”. Therefore, further studies on actual role of the variant in the disease are needed before assuming that a synonymous nucleotide change will have no effect. In addition, some disorders are characterized by highly stereotyped variants that introduce a premature termination codon in the protein [127]: in this case an evaluation of splice impact of a variant classified as “missense” should be considered. It must be remembered that a splice variant causing deletion (or insertion) of one or more amino acids, and then strongly modifying the protein, is more likely to disrupt protein function than a missense variant changing only one amino acid. Care must be also taken to potential in-frame deletion/insertion, which could anyway alter protein critical domains and potentially lead to a gain-of-function effect.

Deep intronic variants are also more difficult to characterize: only a few data on them are available, since they are poorly considered in clinical testing, as the routine analyses do not include these genomic regions. However, the analysis for the presence of such variants should be evaluated when the identification of potentially pathogenic variants in the coding regions and exon/intron boundaries is not effective, and the patient presentation is highly suggestive of a variant in a specific gene [26].

**Figure 6:**
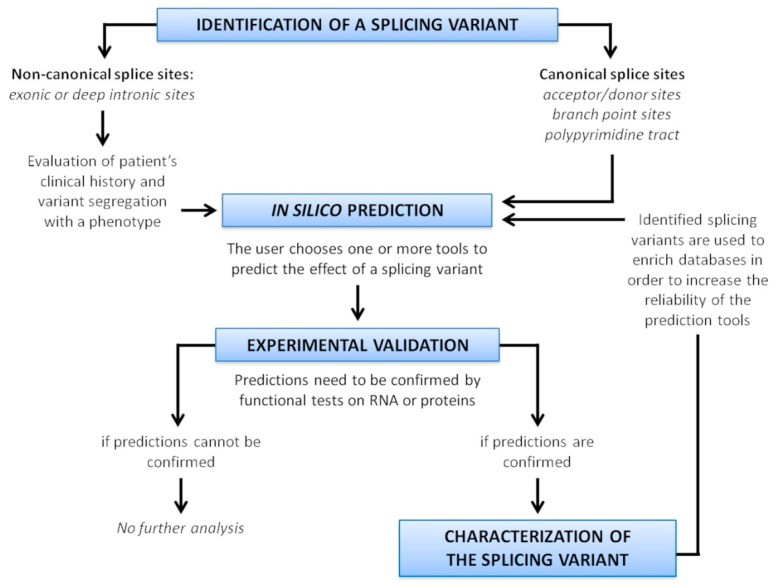
Strategy for splice variant characterization.

Since misclassification of variants have been reported for several diseases [128,129,130,131], an accurate evaluation of potential variant impact on splicing is recommended. A scheme resuming the strategy to characterize splice variants is depicted in Figure 6.

The first step of this analysis is an in silico approach, using tools able to predict the effect of a variant on splicing. The algorithms can have different predictive reliability for different genes, and display their own strengths and weaknesses, therefore it is recommendable to use several tools, or tools incorporating different kind of predictions. Of course, the choice of the tool is crucial: it is necessary to consider the location of the variant in the gene (exonic, intronic, deep intronic), and use a tool able to analyze that specific region. The advent of ML-based approaches has recently increased the predictive power and enhanced the genomic regions considered for the prediction.

As a general rule, when all of the in silico programs agree on the prediction, then this evidence can be considered as supporting. However, though in silico predictions disagree, then this evidence should not be used for variant classification. When prediction algorithms neither predict an impact on a splice consensus sequence nor the creation of a new splice consensus sequence, and the nucleotide position is not conserved over evolution then it is less likely that the variant affects the splicing [4].

Nevertheless, these tools perform predictions, and their use in sequence variant interpretation should be cautious. It is not recommended to use these predictions as the sole basis to make a clinical evaluation. An experimental confirmation is always necessary.

Validation methods can be performed both at gene (mainly through RT-PCR and RNA sequencing) and at protein level (using PTT). Even with the advent of high-throughput methodologies, which allow to fully characterize the transcriptome, conventional experimental RT-PCR, followed by Sanger sequencing, remains the preferred method of analysis for diagnosis of diseases caused by aberrant splicing. For the investigation of a genotype–phenotype correlation in research, RT-PCR may be replaced by microarrays or predominantly by direct sequencing of cDNA, even if RNA-seq is still highly expensive and data interpretation is difficult and troublesome for many laboratories.

## 9. Conclusions

Variants affecting splicing account up to 15% of all point variants causing human genetic disorders. However, recent laboratory evidence has shown that the percentage of these variants seems to be underestimated, since it considers mainly variants involving canonical splice sites. The proper classification of splice variants is essential for the correct diagnosis and genetic counseling. It is currently based on predictive bioinformatics analysis and experimental validation.

Prediction tools and experimental procedures are directly linked to each other. The availability of experimentally validated variants is fundamental for the continuous update of variant databases. All the prediction tools are based on, or learn from, verified variant classification; thus, they may be enhanced only by acquiring more validated experimental data. On the other hand, reliable predictions provided by effective tools may guide variants classification and reduce the number of variants to validate. For this reason, it is important to deepen our knowledge of splicing process, extending the studies outside of the canonical donor and acceptor splice site motifs for splicing mechanisms, in particular in intronic regions. Concurrently, clinical variants databases must be updated with validation results. These advances will be critical to increase the accuracy of bioinformatics predictions and thereby improve the assessment of variant pathogenicity.

## Figures and Tables

**Table 1:** Conserved RNA elements recognized by the splicing machinery.

Splice Sites	Nucleotides
5’ splice site	CAG/GUAAGU
Branch point sequence	YUNAY
Polypyrimidine tract	Y_n_
3’ splice site	NYAG/G

Y = C/U; N = any nucleotide; “n” = number of pyrimidine constituting the polypyrimidine tract.

**Table 3:** Brief summary of the main characteristics of the different methods used in ML.

Method	Main Characteristics
**Regression**	Evaluation of the relationships between input variables and associated outputs and modeling of the relationship between them. Use of continous values. Linear regression: the simplest form, the basic idea is simply finding a line that best fits the data. Multiple linear regression and polynomial regression: focus on non-linear problems Logistic regression: models the probability of an observation to belong to a finite number of classes, typically two (0 and 1).
**Classification**	Finding of a model or function which helps in separating the data into classes based on different parameters. Use of discrete values. Categorization of data under different labels, according to some parameters given in input
**Support Vector Machine (SVM)**	Classification algorithm based on a hyperplane space that linearly separates training observations of different classes and creates a demarcation among the categories. Every unseen sample is classified into one of the classes, depending on the side on which it appears. Data that cannot be separated by a single continuous hyperplane are usually transformed using the kernel functions.
**Decision Tree**	Tree-like support tools used to correspond to a cause and its effect. Each node of the tree represents a test of one or more features of the observation and determines the following nodes to go through. The last nodes of the decision tree, where a decision is taken, are defined leaves of the tree The more nodes are present, the more accurate the decision tree will be. It can use regression or classification algorithms.
**Random Forest**	Combination of multiple decision trees, usually resulting in an improved predictive performance. Use of an “ensemble learning methods” (methods that use multiple learning algorithms to obtain better predictive performance than any of the constituent learning algorithm alone). Efficient modeling of complex and nonlinear data types, overcoming the limitations of Decision Trees. It can use regression or classification algorithms.
**Neural Network (NN)**	Similarity to the biological neural network, it is a collection of connected nodes called “artificial neurons”, which, like in the synapses in a real brain, can transmit information to other nodes or “neurons”. It is a network of mathematical equations. It works on input variables and, by going through a network of equations, transforms them in one or more output variables. Networks are built up of layers, each responsible for a linear transformation, followed by a nonlinear activation function. There are an input layer, one or more hidden layers, and an output layer Generally, more nodes and more layers allow the neural network to make much more complex calculations. It can use regression and classification algorithms, or combinations of them.
**Deep Neural Networks (DNNs)**	NNs with multiple hidden layers between the input and output layers.
**Convolutional Neural Networks (CNN)**	Its architecture is analogous to that of the connectivity pattern of neurons in visual cortex of the human brain. The hidden layers include layers that perform convolutions (in mathematics convolution is a mathematical operation on two functions that produces a third function that expresses how the shape of one is modified by the other).

**Table 5:** Benefits and drawbacks of high-throughput technologies [115].

	Microarray	Rna-Seq
Benefits	Availability of standardized approaches and protocols Low cost procedure (compared to RNA-seq)	Analysis of the whole transcriptome Wide dynamic range Alternative splicing sites can be detected with no biases High specificity and sensitivity
Drawbacks	Analysis only for pre-defined genes Limited dynamic range Absence of specificity for hybridization-based approach Eventual loss of new variants (depending on probe density)	Optimization of the protocols is still poor Expensive procedure compared to microarray Complex data analysis

## Data Availability

Not applicable.
